# Aripiprazol and Hypersexuality: when partial is to much

**DOI:** 10.1192/j.eurpsy.2022.1865

**Published:** 2022-09-01

**Authors:** P. Espada-Santos, J. Facucho-Oliveira, B. Mesquita, A. Fraga, M. Albuquerque, M. Costa, M. Marinho, P. Cintra

**Affiliations:** Hospital de Cascais, Psychiatry, Alcabideche, Portugal

**Keywords:** Aripiprazol, hypersexuality

## Abstract

**Introduction:**

A growing number of published cases has showed that hypersexual behavior may arise with treatment with second-generation antipsychotics, including aripiprazole and olanzapine. Aripiprazole is a second-generation antipsychotic commonly used to treat schizophrenia and bipolar disorder. It has a unique pharmacologic profile acting as a partial agonist of the dopamine D2 receptor, as a partial agonist at the 5-HT1A receptor, and as an antagonist at the 5-HT2A receptor. Literature shows that medication with partial dopaminergic agonistic activity can cause compulsive behaviors, such as pathological gambling, compulsive eating, compulsive shopping, and hypersexuality. Although it is difficult to predict who would develop these behaviors, the literature suggests that patients at a higher risk of developing impulsive behaviors include those with a personal or family history of obsessive-compulsive disorder, impulse control disorder, bipolar disorder, impulsive personality, alcoholism, drug abuse, or other addictive behaviors.

**Objectives:**

Here, we present a case of a 32-year-old male who developed hypersexuality symptoms after receiving aripiprazole as treatment for bipolar disorder.

**Methods:**

We have done a literature review using the MeSH terms Aripiprazole and hypersexuality in the “PubMed”.

**Results:**

After switching Aripiprazole to Risperidone the hypersexuality symptoms started to decrease and got almost complete relief after 2 weeks.

**Conclusions:**

This case highlights the rare hypersexuality side effect that can arise in patients receiving aripiprazole for bipolar disorder treatment. Clinicians should be aware of the increased risk of hypersexuality and other impulsive behaviors as they can significantly impair a patient’s daily functioning.

**Disclosure:**

No significant relationships.

